# Secretion of collagenases by *Saccharomyces cerevisiae* for collagen degradation

**DOI:** 10.1186/s13068-022-02186-y

**Published:** 2022-08-28

**Authors:** Han Xiao, Xiufang Liu, Yunzi Feng, Lin Zheng, Mouming Zhao, Mingtao Huang

**Affiliations:** 1 School of Food Science and Engineering, South China University of Technology, Guangzhou, 510641 China; 2 Guangdong Food Green Processing and Nutrition Regulation Technologies Research Center, Guangzhou, 510650 China

**Keywords:** Recombinant collagenases, *Saccharomyces cerevisiae*, Heterologous expression, Collagen degradation, Synergistic effect

## Abstract

**Background:**

The production and processing of animal-based products generates many collagen-rich by-products, which have received attention both for exploitation to increase their added value and to reduce their negative environmental impact. The collagen-rich by-products can be hydrolyzed by collagenases for further utilization. Therefore, collagenases are of benefit for efficient collagen materials processing. An alternative and safe way to produce secreted collagenases is needed.

**Results:**

Two collagenases from *Hathewaya histolytica*, ColG and ColH, were successfully secreted by the yeast *Saccharomyces cerevisiae.* Compared with the native signal peptide of collagenase, the α-factor leader is more efficient in guiding collagenase secretion. Collagenase secretion was significantly increased in YPD medium by supplementing with calcium and zinc ions. Recombinant collagenase titers reached 68 U/mL and 55 U/mL for ColG and ColH, respectively. Collagenase expression imposed metabolic perturbations on yeast cells; substrate consumption, metabolites production and intracellular cofactor levels changed in engineered strains. Both recombinant collagenases from yeast could hydrolyze soluble and insoluble collagen materials. Recombinant ColG and ColH showed a synergistic effect on efficient collagen digestion.

**Conclusions:**

Sufficient calcium and zinc ions are essential for active collagenase production by yeast. Collagenase secretion was increased by optimization of expression cassettes. Collagenase expression imposed metabolic burden and cofactor perturbations on yeast cells, which could be improved through metabolic engineering. Our work provides a useful way to produce collagenases for collagen resource utilization.

**Graphical Abstract:**

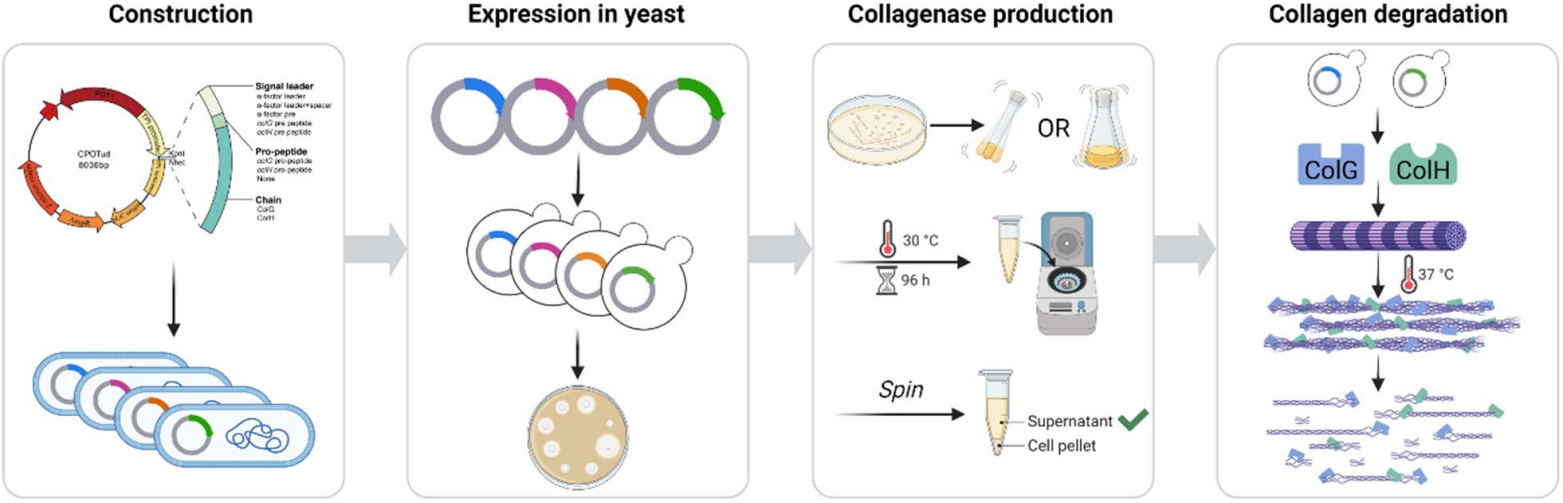

**Supplementary Information:**

The online version contains supplementary material available at 10.1186/s13068-022-02186-y.

## Background

Animal-derived protein consumption has increased in recent decades to support the growing global population and improved quality of life [1]. The production and processing of these animal-based products generates many by-products, such as horns, hooves, skin, bones and scales. These by-products are not edible or cannot be sold on the market. They are either simply processed into low-value products or dumped, which cause environmental problems [2]. However, these by-products can be utilized in a more effective way to reduce environmental burden and their added value can be increased with advanced techniques. Enzymatic processing is an important way to extract bioactive compounds from these by-products. Compared with chemical processes involving extreme conditions (high temperatures, high pressure, corrosive chemicals), enzymatic hydrolysis under mild condition provides a more acceptable way to obtain high-quality bioactive extracts with low toxicity and a low environmental impact [3]. The by-products of livestock, poultry and aquaculture usually contain a large amount of collagen. The bioactive peptides hydrolyzed by collagenases from these by-products have shown antioxidant, antihypertensive and antidiabetic effects. Therefore, they have broad application in the food, pharmaceutical and cosmetic industries [4–7].

Collagenases are enzymes capable of cleaving native collagen under physiological conditions [8]. *Hathewaya histolytica* collagenases are the most well studied microbial collagenase and are recognized as the gold standard for comparing newly found collagenolytic enzymes [9]. Currently, *H. histolytica* collagenases play an important role in medical treatment for wound healing, burns, keloid, cellulite and some diseases including Dupuytren’s disease, intervertebral disc herniation, urologic disease and others [10–12]. There are two classes of collagenases secreted by *H. histolytica*, and they are classified based on their hydrolytic activity against natural collagen substrates and a specific synthetic substrate FALGPA (N-[3-(2-furyl) acryloyl]-Leu-Gly-Pro-Ala) [13–15]. Class I collagenase, encoded by *colG* shows high activity against collagen and moderate activity against FALGPA, whereas class II collagenase, encoded by *colH* has lower activity against collagen and higher activity against FALGPA [16–19].

Although collagenases can be produced by *H. histolytica*, other unwanted components, such as clostripin, trypsin and neutral protease, are also found in crude collagenases and cause deterioration of crude collagenase during storage [20]. High-purity collagenase requires tedious purification steps, during which the production cost increases and collagenase activity may reduce. Furthermore, *H. histolytica* is pathogenic in humans, which hinders application of its collagenases in the field considering safety as a priority. Researchers have produced recombinant *H. histolytica* collagenases by using *Escherichia coli* expression system [21, 22]. Although both ColG and ColH were expressed successfully, these collagenases were of intracellular expression and sometimes encountered insolubility problems. Therefore, an alternative and safe way to produce secreted collagenases is needed. The yeast *Saccharomyces cerevisiae* is one of the most popular hosts for protein production, as it possesses a protein secretory pathway, is easily manipulated and has tolerance to industrial conditions [23]. Considering its GRAS status and widespread use in the food industry, supernatant containing recombinant enzymes from yeast cultures can possibly be applied in downstream processes directly. However, there are few reports on the heterologous expression of collagenase from *H. histolytica* in eukaryotes.

In this study, we successfully expressed the *H. histolytica* collagenases in secreted form by *S. cerevisiae*. Culture conditions and expression cassettes were optimized for efficient collagenase production. Influences on physiological traits of yeast cells by collagenase expression were also characterized. Collagen could be degraded by recombinant collagenases from yeast, and the synergistic effect of two recombinant collagenases was revealed for efficient collagen digestion. Our study provides a useful way to produce collagenases for better utilization of collagen resources.

## Results and discussion

### Recombinant collagenase expression in *S. cerevisiae*

**Fig. 1:**
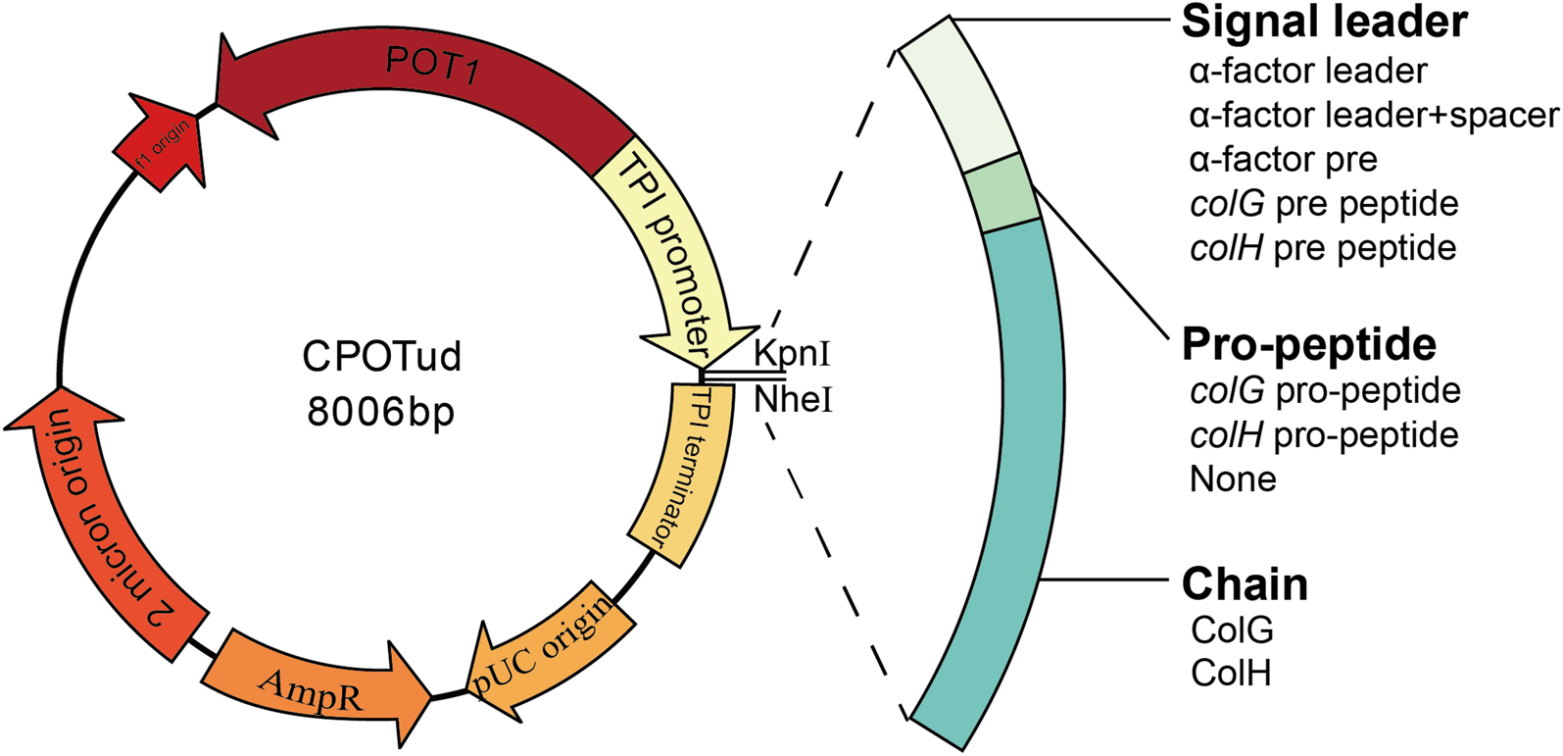
Construction scheme of plasmids for recombinant expression of collagenases. α-factor leader: the leader sequence of the α-factor mating pheromone; α-factor leader + spacer: α-factor leader with a spacer sequence (EEGEPK) replacement of EAEA at the C-terminus; α-factor pre: the pre region of α-factor leader

In this study, both collagenase ColG (UniProt Entry: Q9X721) and collagenase ColH (UniProt Entry: Q46085) from *H. histolytica* were expressed in *S. cerevisiae*. The yeast strain *S. cerevisiae* B184M [24], a UV mutant with superior protein secretion, was chosen as the host strain for the heterologous expression of ColG and ColH. A widely used signal peptide in yeast, the α-factor leader, was used for directing secretion. Firstly, codon-optimized genes *colG* and *colH* fused with signal leader sequences were inserted into the vector CPOTud, resulting in pCP_G01 and pCP_H01 (Fig. 1 and Table 1), respectively. Both pCP_G01 and pCP_H01 were transformed to strain B184M, and strains BG01 and BH01 were obtained. Then strains BG01 and BH01 were tested for their collagenase expression in YPD medium, but little collagenase activity was detected in the supernatant.

**Table 1:** Strains and plasmids used in this study

Strains and plasmids	Description	Reference or source
*Saccharomyces cerevisiae* strain		
CEN.PK 530-1C	*MATa tpi1(41–707)::loxP-KanMX4-loxP*	Overkamp et al. [34]
B184M	UV-mutated strain derived from CEN.PK 530.1C	Huang et al. [7]
B0	B184M/CPOTud	This study
BG01	B184M/pCP-G01	This study
BG02	B184M/pCP-G02	This study
BG03	B184M/pCP-G03	This study
BG04	B184M/pCP-G04	This study
BG05	B184M/pCP-G05	This study
BG06	B184M/pCP-G06	This study
BG07	B184M/pCP-G07	This study
BH01	B184M/pCP-H01	This study
BH02	B184M/pCP-H02	This study
BH03	B184M/pCP-H03	This study
BH04	B184M/pCP-H04	This study
BH05	B184M/pCP-H05	This study
Plasmid		
pUC57-Mini_G	Vector pUC57-Mini with *colG* gene (*colG* pre-peptide‐*colG* pro-peptide‐*colG* chain) from *Clostridium histolyticum*	GenScript co. Ltd
pUC57-Mini_H	Vector pUC57-Mini with *colH* gene (*colH* pre-peptide‐*colH* pro-peptide‐*colH* chain) from *Clostridium histolyticum*	GenScript co. Ltd
CPOTud	2 μm, AmpR, *TPI1*p-(gene of interest)-*TPI1*t, *POT1* gene from *S. pombe* as a selection marker	Liu et al. [40]
pAlphaAmyCPOT	CPOTud‐(α-factor leader + spacer‐amylase gene)	Liu et al. [40]
pCP_G01	CPOTud‐(α-factor leader + spacer‐*colG* pro-peptide‐*colG* chain)	This study
pCP_G02	CPOTud‐(α-factor leader‐*colG* pro-peptide‐*colG* chain)	This study
pCP_G03	CPOTud‐(α-factor pre‐*colG* pro-peptide‐*colG* chain)	This study
pCP_G04	CPOTud‐(α-factor leader + spacer‐*colG* chain)	This study
pCP_G05	CPOTud‐(α-factor leader-*colG* chain)	This study
pCP_G06	CPOTud‐(α-factor pre‐*colG* chain)	This study
pCP_G07	CPOTud‐(*colG* pre-peptide‐*colG* pro-peptide‐*colG* Chain)	This study
pCP_H01	CPOTud‐(α-factor leader + spacer‐*colH* pro-peptide‐*colH* chain)	This study
pCP_H02	CPOTud‐(α-factor leader‐*colH* pro-peptide‐*colH* chain)	This study
pCP_H03	CPOTud‐(α-factor leader + spacer-*colH* chain)	This study
pCP_H04	CPOTud‐(α-factor leader‐*colH* chain)	This study
pCP_H05	CPOTud‐(*colH* pre-peptide‐*colH* pro-peptide‐*colH* chain)	This study

**Fig. 2:**
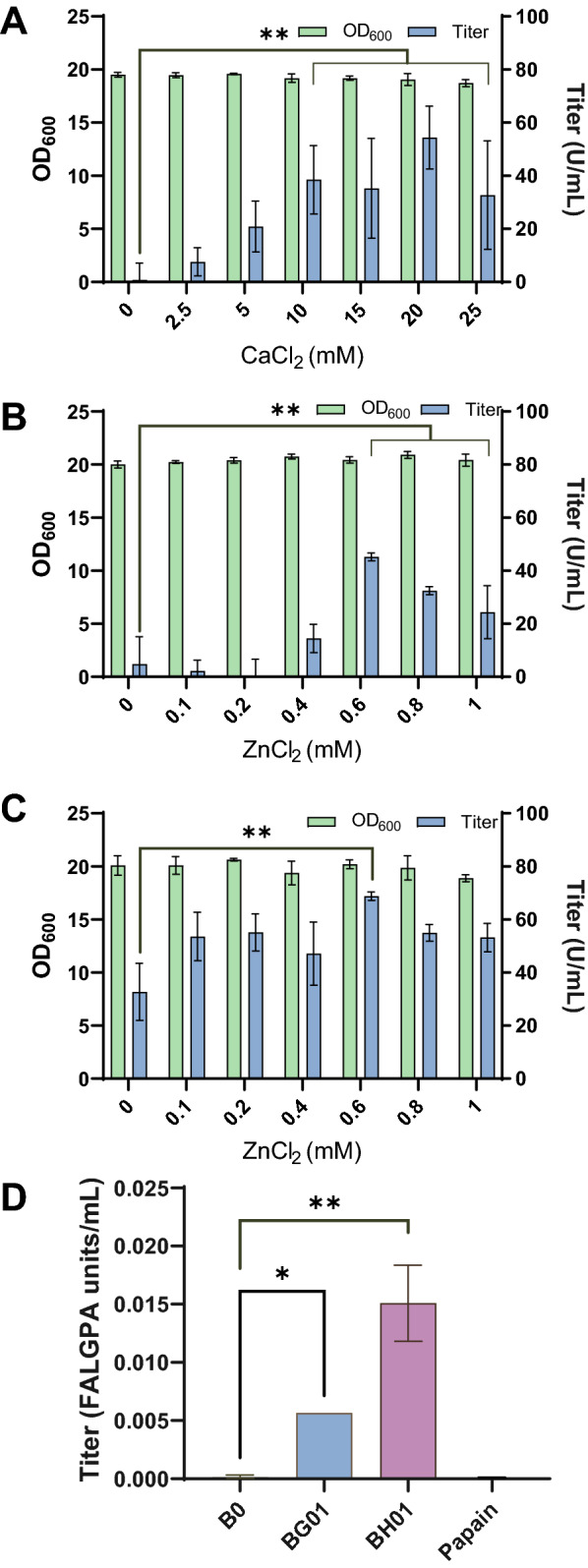
Optimization of metal ion concentration in culture medium. **A** YPD medium supplemented with different concentrations of CaCl_2_; **B** YPD medium supplemented with different concentrations of ZnCl_2_; **C** YPD medium containing 10 mM CaCl_2_ and supplemented with different concentrations of ZnCl_2_; **D** enzymatic assay of collagenase in supernatant using FALGPA (N-(3-[2-furyl]acryloyl)-Leu-Gly-Pro-Ala) as the substrate. The strain B0 harboring the empty plasmid CPOTud was used as control. Papain: 1 mg/mL papain solution. * P < 0.05; ** P < 0.01

Similar to other metalloproteases, ColG and ColH also require Zn^2+^ for catalytic reaction and are stabilized by Ca^2+^ [25–29]. We speculated that YPD medium without enough Ca^2+^ and Zn^2+^ ions would repress collagenase activity and supplementation of Ca^2+^ and Zn^2+^ to the medium would help collagenase expression. Therefore, we cultured strain BG01 in YPD medium with various concentrations of CaCl_2_ and ZnCl_2_, and collagenase activity did increase significantly (Fig. 2). When CaCl_2_ and ZnCl_2_ were solely added to YPD medium, the collagenase ColG activity was higher in the range of 10 ~ 20 mM CaCl_2_ (Fig. 2A) and 0.6 mM ZnCl_2_ was better (Fig. 2B). To reduce potential negative effects of excessive ions, 10 mM CaCl_2_ was selected for combinational addition with ZnCl_2._ As expected, simultaneous addition of CaCl_2_ and ZnCl_2_ increased collagenase ColG activity further (Fig. 2C). The optimal concentration of ZnCl_2_ in the presence of 10 mM CaCl_2_ for collagenase ColG expression was 0.6 mM, which was consistent with the result of adding ZnCl_2_ alone. Then, supplementation of CaCl_2_ and ZnCl_2_ to the medium was also tested for ColH expression. Like ColG, ColH achieved its optimal expression in YPD medium with 10 mM CaCl_2_ and 0.6 mM ZnCl_2_ (Additional file 1: Fig. S1). These results confirm the importance of metal ions for heterologous expression of collagenase by yeast, and provided practical information for expression of other metalloproteases. It should be pointed out that selection of metal ions and optimal metal ion concentration depend on the properties of the metalloproteinase and host strain.

Besides gelatin, the substrate FALGPA, a specific substrate for collagenase, was used to detect collagenase activity of ColG and ColH. Both BG01 and BH01 showed FALGPA hydrolysis activity, compared with no FALGPA activity by papain (Fig. 2D). This result again confirmed our success in heterologous secreted expression of ColG and ColH in yeast cells. Furthermore, BH01 had higher FALGPA hydrolysis activity than that of BG01. This result agreed with previous reports that class II collagenase (here, ColH) is more efficient in hydrolysis of FALGPA than class I collagenase [14].

### Optimization of collagenase expression

**Fig. 3:**
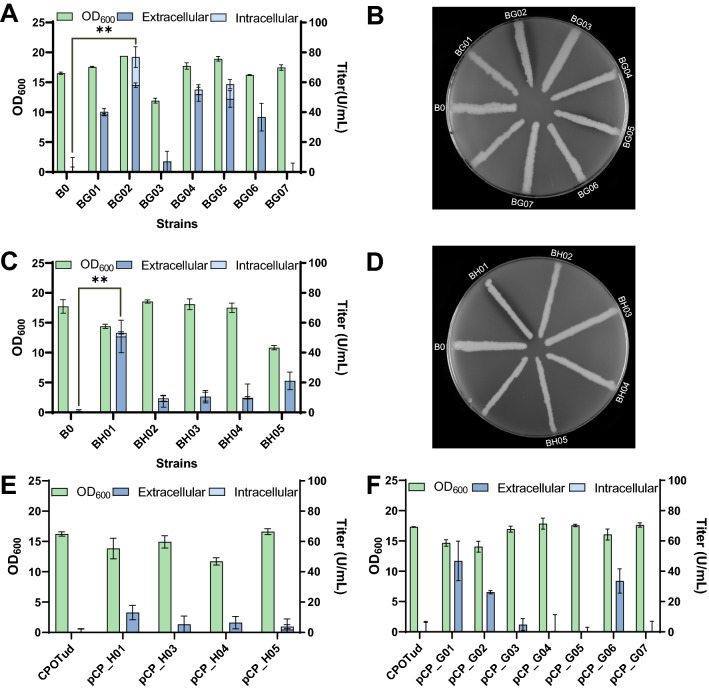
Collagenase secretion by yeast strains harboring plasmids with different expression cassettes. **A** Cell growth and collagenase secretion by B184M yeast strains expressing ColG, ** P < 0.01; **B** halo-plate assay for yeast strains expressing ColG; **C** cell growth and collagenase secretion by B184M yeast strains expressing ColH, ** P < 0.01; **D** halo-plate assay for yeast strains expressing ColH; **E** cell growth and collagenase secretion by CEN.PK 530-1C yeast strains expressing ColG; **F** cell growth and collagenase secretion by CEN.PK 530-1C yeast strains expressing ColH; strain B184M was derived from the strain CEN.PK 530-1C by UV mutation

After proving secretion of collagenase in yeast, we attempted to optimize secretion levels through altering the expression cassette. Therefore, we changed the signal leader in pCP_G01 to the native collagenase pre-peptide or α-factor pre. The ColG pro-peptide was also removed in some of these cassettes as detailed in Fig. 1 and Table 1. These new plasmids for ColG expression were constructed and transformed into strain B184M (Table1). ColG activity was different in these strains, among which strain BG02 had the highest collagenase titer and yield (Fig. 3A and Additional file 1: Fig. S2A). Distinctive halos were formed around BG02 colonies on the agar plate with 1% skimmed milk (Fig. 3B). Similarly, plasmids with different expression cassettes of ColH were constructed and transformed into strain B184M. Strain BH01 showed the highest ColH collagenase titer and yield among ColH expression strains (Fig. 3C and D, Additional file 1: Fig. S2B). Previously, it was reported that the pro-peptide is not essential for *H. histolytica* collagenases as intracellular expression by *E. coli* [30]. However, secreted expression *H. histolytica* collagenases in yeast was affected by the pro-peptide, especially for ColH. As pro-peptides can either directly assist or accelerate the protein folding process [31–33], it may provide benefits for proper folding of collagenases in yeast cells. However, details about the role of collagenase pro-peptides in yeast expression require further elucidation. As mentioned above, we used a mutant yeast strain B184M for collagenase expression. We subsequently tested the effect of collagenase expression in a wild-type yeast strain. Plasmids were transformed to the wild-type strain CEN.PK 530-1C [34], and collagenase activities were quantified for these strains (Fig. 3E and F). Although collagenase activity was detectable in the CEN.PK 530-1C strain with certain plasmids, the general collagenase production level by strain CEN.PK 530-1C was much lower than that of strain B184M. In previous studies, researchers showed the importance of promoter strength and regulation for gene expression [35, 36], To discover whether collagenase secretion was affected by using different promoters, we replaced the promoter–terminator *TPI1*p-*TPI1*t of the vector CPOTud with two other pairs *PGK1*p-*CYC1*t and *TEF1*p-*ADH1*t for ColG expression. Although ColG activity changed by using different promoters, no increased ColG activity was found. The promoter *TPI1*p was still the best for collagenase expression (Additional file 1: Fig. S3), and thus was used in the subsequent study. Achieving higher collagenase secretion levels may be possible through testing more combinatorial promoter–terminator pairs for fine-tuning of collagenase expression in future studies.

**Fig. 4:**
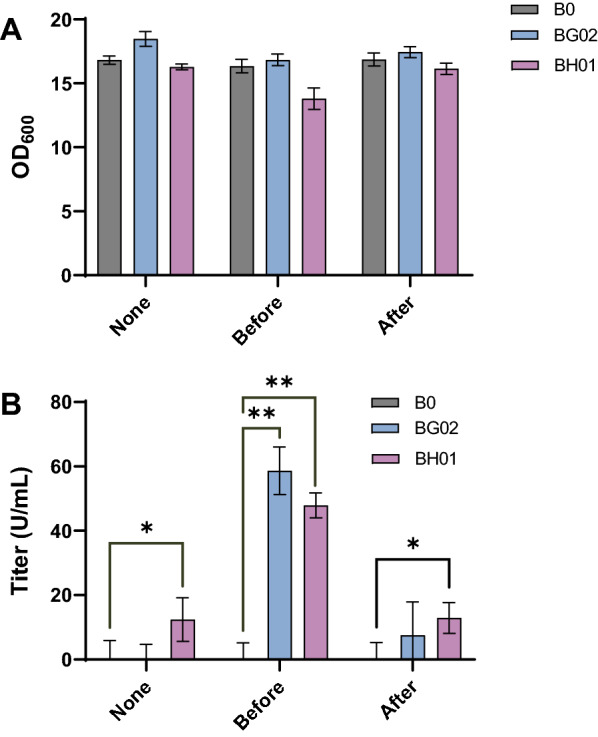
Metal ions involved in incorporation to collagenases during production in yeast cells instead of in vitro assembly. **A** Cell growth; **B** collagenase activity. None: strains B0, BG02 and BH01 were cultured in YPD medium without supplementation of CaCl_2_ and ZnCl_2_. Before: B0, BG02 and BH01 were cultured in YPD medium supplemented with 10 mM CaCl_2_ and 0.6 mM ZnCl_2_. After: B0, BG02 and BH01 were cultured in YPD medium; when fermentation was finished, CaCl_2_ and ZnCl_2_ were added to the culture at a final concentration of 10 mM and 0.6 mM, respectively. * P < 0.05; ** P < 0.01

Furthermore, we evaluated collagenase activity in case of adding metal ions to YPD medium before and after cultivation. As shown in Fig. 4, the collagenase activity in conditions of supplying metal ions before cultivation was much higher than after fermentation. Compared with the control (medium without additional metal ions), collagenase activities had little change when adding metal ions to medium after fermentation. This result indicates that metal ions were incorporated into collagenases during intracellular biological processing within yeast cells instead of in vitro assembly.

### Evaluation of yeast cell growth and collagenases expression

**Fig. 5:**
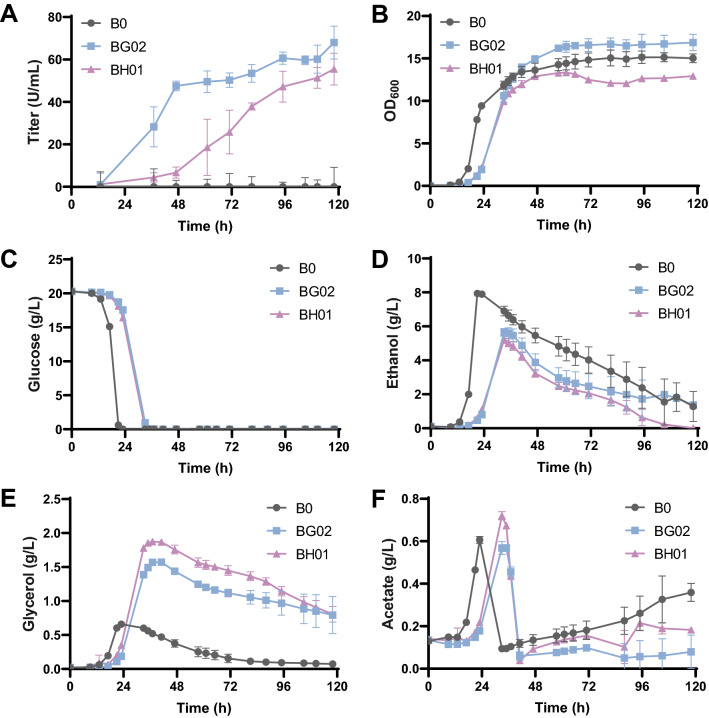
Batch cultivation of strains BG02, BH01 and the control strain B0. **A** Collagenase production; **B** cell growth; **C** glucose consumption; **D** ethanol production; **E** glycerol production; **F** acetate production. YPD medium with 10 mM CaCl_2_ and 0.6 mM ZnCl_2_ was used for cell cultivation

In order to investigate potential metabolism changes of yeast cells when expressing collagenases, strains BG02 and BH01 together with the control strain B0 were characterized in batch fermentation (Fig. 5). Although both strains BG02 and BH01 grew slower than the control strain B0 (Fig. 5B), the final biomass of strains BG02 and BH01 differed. Strain BH01 had lower final biomass than the control strain B0, yet the final biomass of strain BG02 was approximately 12% higher than that of strain B0. Compared with strain B0, strain BG02 and BH01 showed slower glucose consumption (Fig. 5C). Changes in biomass and glucose consumption suggest that collagenase expression imposed metabolic burden on yeast cells. Interestingly, the collagenase production process of ColG was different from that of ColH, though the collagenase activity and collagenase yield at the end of fermentation was similar for ColG and ColH (Fig. 5A and Additional file 1: Fig. S4). ColG reached a relatively high level earlier (at 48 h) and then increased slowly. ColH secretion lagged behind. Relatively little ColH was detected at 48 h and ColH collagenase activity started increasing afterwards. We also found that strains BG02 and BH01 produced less ethanol than strain B0 (Fig. 5D). In contrast, glycerol production by BG02 and BH01 was higher than that by strain B0 (Fig. 5E). These results indicate that intracellular energy demand and redox state was affected by expression of the collagenases. As a result of ColG expression, strain BG02 had higher ATP level and GSH (Additional file 1: Fig. S5A and S5B). This might explain the higher final biomass for strain BG02. Reduced NADH/NAD^+^ ratio and NADPH/NADP^+^ ratios were found in strain BG02 and BH01 (Additional file 1: Fig. S5C and S5D). This confirmed the metabolic impact of redox cofactor perturbations by expression of collagenases. Biosynthesis of amino acids for protein synthesis is linked with NADPH and NADH, which can be oxidized via mitochondrial respiration or glycerol production to maintain redox homeostasis [37]. Thus, supply of amino acids may be a direct way of decreasing the influence of recombinant protein production [38, 39]. Moreover, NADPH plays an important role in supporting ER normal function, which is essential for secreted protein folding [40]. Therefore, increasing the NADPH level or promoting additional NADH conversion to NADPH may also have a positive impact on recombinant proteins production.

### Substrate degradation by recombinant collagenases

**Fig. 6:**
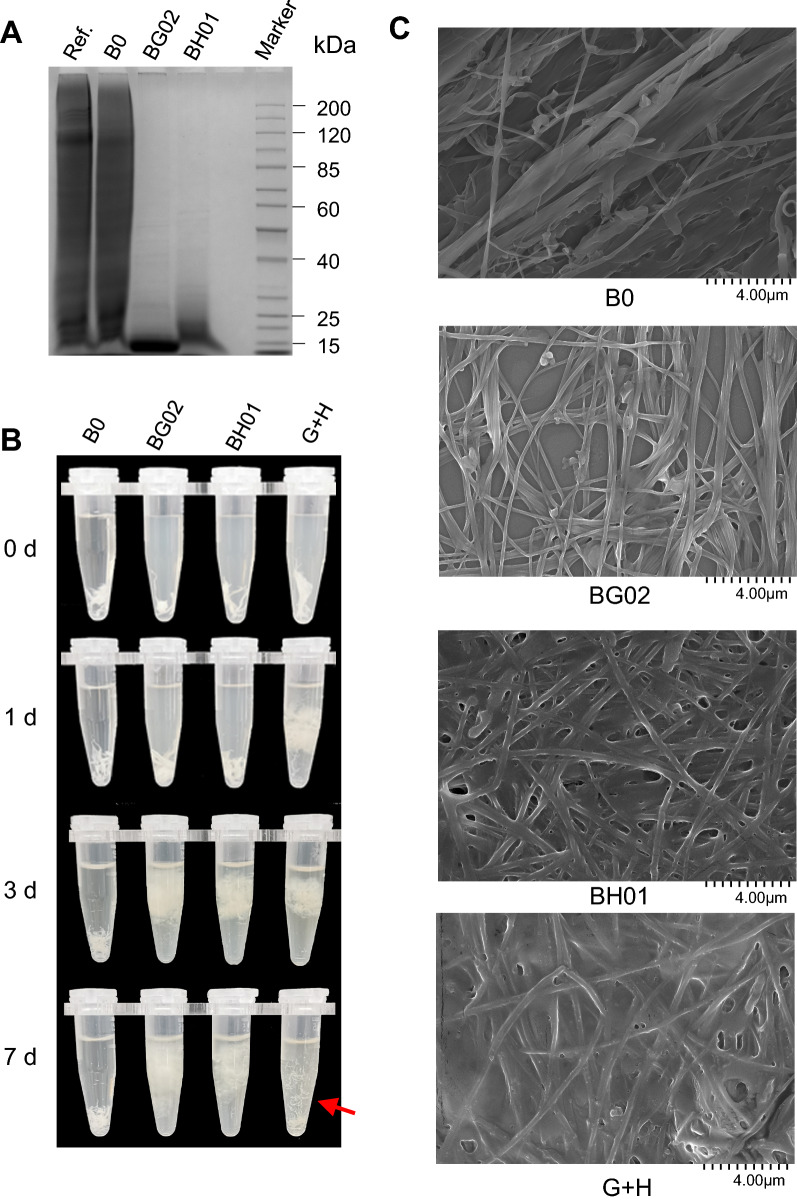
Collagen substrate degradation by recombinant collagenases. **A** SDS-PAGE analysis of gelatin digestion by recombinant collagenases during cell cultivation. Strains were cultivated in YPD medium with 10 mM CaCl_2_, 0.6 mM ZnCl_2_ and 1% gelatin at 30 °C for 96 h, then supernatant was used for analysis. Ref.: YPD medium with 10 mM CaCl_2_, 0.6 mM ZnCl_2_ and 1% gelatin. **B** Collagen hydrolysis by recombinant collagenases. Strains were cultivated in YPD medium with 10 mM CaCl_2_ and 0.6 mM ZnCl_2_ at 30 °C for 96 h for collagenase production. The supernatant was collected and replaced with 50 mM Tris–HCl (pH = 7.5) by using an Amicon^®^ Ultra filter (50 kDa). Subsequently, collagen was added to tubes and incubated at 37℃. G + H: supernatants from BG02 and BH01 were mixed with a ratio of 1:1. **C** Differences between recombinant ColG and ColH in collagen degradation were revealed at microscopic structure level by using a scanning electron microscope (SEM) with 20,000 × magnification. Day 1 degradation samples were used for SEM analysis

To test the degradation of collagen materials by recombinant collagenases, we added gelatin to culture medium. After fermentation, supernatants were analyzed by SDS-PAGE then stained with Coomassie brilliant blue, by which gelatin would be labeled. As shown in Fig. 6A, gelatin was successfully degraded by strains BG02 and BH01 during cultivation. Strains harboring plasmids with different collagenase cassettes were also tested for gelatin degradation (Additional file 1: Fig. S6), the result of which was consistent with their collagenase activity (Fig. 3A and C). As class I collagenases and class II collagenases have different hydrolytic preferences towards collagen [17, 41–43], we inferred a synergistic effect by ColG and ColH [44]. Therefore, we mixed supernatants of strains BG02 and BH01 in different proportions, and measured collagenase activity using gelatin as a substrate. A slight synergistic effect was found in the mixture of ColG as majority (Additional file 1: Fig. S7). As gelatin is a degraded form of collagen, the synergistic effect of BG02 and BH01 might be more significant for integral collagen. Hence, bovine insoluble collagen was used for digestion test by recombinant collagenases. Both recombinant collagenase ColG and collagenase ColH were able to degrade collagen, and ColG was more effective in digestion with a swelling manner. Furthermore, ColG and ColH did act synergistically in hydrolysis of collagen (Fig. 6B). This is consistent with the fact that ColG prefers to degrade collagen in a processive manner, while ColH tends to bind to these swollen areas for hydrolysis [25, 43]. Degradation of collagen fibrils by recombinant ColG and ColH was also revealed by using a scanning electron microscope (SEM) (Fig. 6C, Additional file 1: Fig. S8). Compared with the control, the collagen substrate degraded by ColG and ColH was loose and the three-dimensional helix structure was much impaired. The sample degraded by ColG + ColH showed even more viscous and looser (Fig. 6C). These results support the effective degradation of collagen by recombinant collagenases from microscopic aspects.

## Conclusions

Two collagenases ColG and ColH were successfully secreted by *S. cerevisiae*. Supplementation of Ca^2+^ and Zn^2+^ was crucial for high-level collagenase production. Collagenase production was increased by optimization of expression cassettes. Collagenase expression imposed metabolic burden and cofactor perturbations on cells. The production processes of collagenases were different; ColG production reached a plateau earlier while ColH production had a lag phase. This provided clues for selecting potential targets to engineer yeast strains for efficient collagenase production in the future. Recombinant collagenases degraded collagen and the synergistic effect of collagenases contributed to efficient collagen digestion. To the best of our knowledge, this is the first time a synergistic effect for collagen degradation by recombinant collagenases has been shown. Expression of collagenase in secreted form can simplify downstream processing, compared with intracellular expression. Considering the GRAS status of *S. cerevisiae*, the supernatant containing collagenases could possibly be used as crude enzyme solution directly and adapted with collagen material processing easily. Therefore, our findings are beneficial for effective utilization of collagen resources.

## Materials and methods

### Construction of strains and plasmids

The strains and plasmids used in this study are listed in Table 1. The primers used in this study are listed in Additional file 1: Table S1. The protein sequences of ColG (UniProt Entry: Q9X721) and ColH (UniProt Entry: Q46085) were obtained from UniProt database. Corresponding coding DNA sequences for ColG and ColH were codon-optimized and synthesized by GenScript. Standard methods in molecular biology, including PCR, fusion PCR, enzyme digestion, ligation, etc., were used for DNA fragment amplification and plasmid construction [45]. The PCR was performed on a thermal cycler (T100, Bio-Rad, USA) using 2 × Phanta Max Master Mix (Vazyme, China), following the manufacturer’s instructions.

For construction of plasmids via enzyme digestion and ligation, the CPOTud, a 2-micron plasmid of *S. cerevisiae* with a copy number of 40–60 per haploid cell, was linearized by Kpn I and Nhe I. Primer pair AF/ARproG, AF/ARWproG, AF/ARG, AF/ARWG, AF/ARproH, AF/ARWproH, AF/ARH, AF/ARWH were used to amplify signal peptide with ColG or ColH homologous arms, using the plasmid pAlphaAmyCPOT as template. Primer pair proGF/GR, WproGF/GR, AGF/GR, WGF/GR, proHF/HR, WproHF/HR, AHF/HR, WHF/HR were used to amplify ColG or ColH chain, using the plasmid pUC57-Mini_G or pUC57-Mini_H as template. Then the signal peptide fragments and the ColG or ColH chains were fused together by overlapping fusion PCR, resulting in gene fragments G01, G02, G03, G04, G05, H01, H02, H03 and H04. Gene fragment G06 was constructed by two rounds of PCR using primer pair PG/GR and AP/GR. Gene fragment G07 was amplified from vector pUC57-Mini_G using primer GF and GR, while H05 was amplified from vector pUC57-Mini_H using primer HF and HR. All of these gene fragments were inserted into CPOTud to construct plasmids pCP_G01, pCP_G02, pCP_G03, pCP_G04, pCP_G05, pCP_G06, pCP_G07, pCP_H01, pCP_H02, pCP_H03, pCP_H04 and pCP_H05. *Escherichia coli* DH5α was used to construct and propagate recombinant plasmids. Plasmids were introduced into *E. coli* via the standard heat shock method, and *S. cerevisiae* via the LiAc/SS carrier DNA/PEG method [46].

### Media and culture conditions

*E. coli* DH5α was cultivated in LB medium (5 g/L yeast extract, 10 g/L tryptone, and 10 g/L NaCl) at 37 ℃, 250 rpm. A final concentration of 100 mg/L ampicillin was supplemented to LB medium for plasmid selection. Yeast strains B184M and CEN.PK 530.1C were cultivated in YPE medium (10 g/L yeast extract, 20 g/L peptone, 10 g/L ethanol and 0.5 g/L glucose) on a shaker (ZQZY-CF9.9, Shanghai Zhichu Instruments Co., Ltd., China) at 30 ℃, 200 rpm for cell proliferation. Yeast strains transformed with expression plasmids were generally cultivated at 30℃ in YPD medium (10 g/L yeast extract, 20 g/L peptone, and 20 g/L glucose). For collagenase production, 10 mM CaCl_2_ and 0.6 mM ZnCl_2_ were added to YPD medium, and yeast cells were cultivated at 30℃, 200 rpm.

### Collagenase activity assay

After being cultivated at 30 ℃ for 96 h, cell cultures were centrifuged at 12,000 × g for 3 min. The supernatant was used for extracellular collagenase measurements. Cell pellets were washed twice with distilled water and then resuspended into 0.1 M PBS (pH = 7.5). The cell suspension was transferred into a 2-mL tube containing 0.7 g of 0.5-mm glass beads. Cell lysis was processed in a Bioprep-24R homogenizer (Allsheng, China) at 10 °C, 7 m/s for 1 min twice with an interval of 2 min. Cell debris was removed by centrifugation, and the supernatant fraction was used for intracellular collagenase quantification.

Collagenase activity measurement was modified from that described by Zhang et al. [47]. Briefly, the substrate gelatin was prepared by dilution with reaction buffer (50 mM Tris–HCl with 5 mM CaCl_2_ and 1 μM ZnCl_2_, pH = 7.5) to 2 g/L. A 20 μL sample of supernatant was added to 480 μL gelatin solution and the digestion reaction was conducted at 37℃ for 30 min. Quench buffer containing 12% (w/v) PEG 6000 and 25 mM EDTA was used to stop the reaction. For color development, 100 μL diluted reaction solution was mixed with 500 μL ninhydrin reagent and heated at 80℃ for 10 min. Once the tubes were cooled, an additional 600 μL H_2_O was added and mixed well. The absorbance of reaction mixture was measured at 570 nm with a spectrophotometer. One unit of collagenase activity was defined as the amount of enzyme that liberated 1 μg glycine per minute under the conditions used.

Halo-plate assays were carried out on plates with YPD medium containing 10 mM CaCl_2_, 0.6 mM ZnCl_2_, 1% skimmed milk and 2% agar. Yeast strains were grown on plates with this medium at 30℃ for 4 days and then the plate was incubated at 37℃ for 3 days. Collagenase production was indicated by the formation of clearly visible halos around colonies.

### Substrate degradation test

Gelatin degradation: strains were grown in YPD medium with 10 mM CaCl_2_, 0.6 mM ZnCl_2_ and 1% gelatin at 30 ℃ for 96 h. After cultivation, the supernatant was collected by centrifugation, mixed with SDS-PAGE Sample Loading Buffer and heated at 100℃ for 10 min. Then samples were loaded onto SurePAGE™ PAGE Gel (4–12%) (GenScript, China) and stained with Coomassie brilliant blue after gel electrophoresis.

Collagen degradation: strains were cultivated in YPD medium with 10 mM CaCl_2_ and 0.6 mM ZnCl_2_ at 30℃ for 96 h. The supernatant was collected by centrifugation filtered by Amicon^®^ Ultra (30 kDa) and then replaced with 50 mM Tris–Cl buffer (pH = 7.5). Collagen was added to the prepared supernatant solution and inoculated at 37℃ for hydrolysis by collagenases. The collagen fibrils were taken from the incubated tubes after 24 h and placed on silicon wafers to dry naturally before being bonded to the conductive adhesive. They were examined by a scanning electron microscopy (SEM) at an accelerating voltage of 10.0 kV after gold sputter coating using a Hitachi FESEM SU8220 system (Hitachi, Japan).

### Metabolites and cofactors analysis

Strains in seed cultures were grown overnight to reach the late exponential phase. Then seed cultures were inoculated to 50 mL YPD medium with 10 mM CaCl_2_ and 0.6 mM ZnCl_2_ with an initial OD_600_ of 0.005. Samples were taken at different time points for analysis. The concentration of metabolites (glucose, ethanol, glycerol, etc.) in the culture was measured as described by Huang et al. [48]. 20μL supernatant was loaded to an Aminex HPX-87H column (Bio-Rad, USA) on a Nexera XR HPLC system (Shimadzu, Japan) and the mobile phase was 5 mM H_2_SO_4_ with a flow rate of 0.6 mL/min at 45 °C.

Intracellular ATP, NAD(H), NADP(H) and GSH were determined by using ATP assay kit (Cat No. BC0300, Solarbio, China), NAD(H) assay kit (Cat No. BC0310, Solarbio, China), NADP(H) assay kit (Cat No. BC1100, Solarbio, China) and GSH assay kit (Cat No. BC1170, Solarbio, China), respectively, according to the manufacturer’s protocols.

## Supplementary Information

**Additional file 1: Figure S1.** Optimization of metal ions concentration in culture medium for ColH expression. **Figure S2.** (A) Biomass and collagenase yield of yeast strains B184M expressing ColG; (B) Biomass and collagenase yield by yeast strains B184M expressing ColH. **Figure S3.** Collagenase expression under control by different protomers and terminators, which replaced the promoter and terminator on the plasmid pCP_G02. Strain B184M was used as a host strain. **Figure S4**. Collagenase yield in batch cultivation of strains BG02, BH01 and the control strain B0. **Figure S5.** Intracellular cofactor level changed in collagenase expression strains. **Figure S6.** SDS-PAGE analysis of gelatin digestion by recombinant collagenases during cell cultivation. **Figure S7.** Collagenase activity measurement for the mixture of recombinant ColG and ColH. **Figure S8.** Differences between recombinant ColG and ColH in collagen degradation were revealed at microscopic structure level by using a scanning electron microscope (SEM) with 4000× magnification. **Table S1.** Primers used in this study

## Data Availability

All data generated or analyzed during this study are included in this published article and its Additional file 1: information files.
