# Correction: Secretion of collagenases by *Saccharomyces cerevisiae* for collagen degradation

**DOI:** 10.1186/s13068-023-02372-6

**Published:** 2023-08-01

**Authors:** Han Xiao, Xiufang Liu, Yunzi Feng, Lin Zheng, Mouming Zhao, Mingtao Huang

**Affiliations:** 1 School of Food Science and Engineering, South China University of Technology, Guangzhou, 510641 China; 2 Guangdong Food Green Processing and Nutrition Regulation Technologies Research Center, Guangzhou, 510650 China

**Correction: Biotechnology for Biofuels and Bioproducts (2022) 15:89** **https://doi.org/10.1186/s13068-022-02186-y**

Following publication of the original article [1], the author noticed an error in Additional file 1: Fig. S5 within the additional information. The error in the figure was caused by an incorrect conversion parameter used in the calculation formula. The authors have reanalyzed the data using the correct conversion calculation. The corrected Additional file 1: Fig. S5 has now been published with this correction. The authors also state that the correction does not affect the discussion or conclusions and that they sincerely apologize for the unintentional errors.

## Supplementary Information

**Additional file 1: Figure S5.** Intracellular cofactor level changed in collagenase expression strains.
