# Supplementary material for: Correction: Secretion of collagenases by Saccharomyces cerevisiae for collagen degradation
Source: Biotechnol Biofuels Bioprod. 2023 Aug 1;16:120. doi: 10.1186/s13068-023-02372-6 (PMC10394854; doi:10.1186/s13068-023-02372-6)
Supplement: Supplementary file 1 — Additional file 1: Figure S5. Intracellular cofactor level changed in collagenase expression strains. [file 13068_2023_2372_MOESM1_ESM.pdf]

Supplementary material

Secretion of collagenases by *Saccharomyces cerevisiae* for collagen degradation

Han Xiao [a, b], Xiufang Liu [a, b], Yunzi Feng [a, b], Lin Zheng [a, b], Mouming Zhao [a, b] and Mingtao Huang [a, b, *]

[a] School of Food Science and Engineering, South China University of Technology, Guangzhou, 510641, China

[b] Guangdong Food Green Processing and Nutrition Regulation Technologies Research Center, Guangzhou, 510650, China

* Corresponding author. E-mail address: huangmt@scut.edu.cn

This file contains:

Supplementary Figure S1-S8

Supplementary Table 1

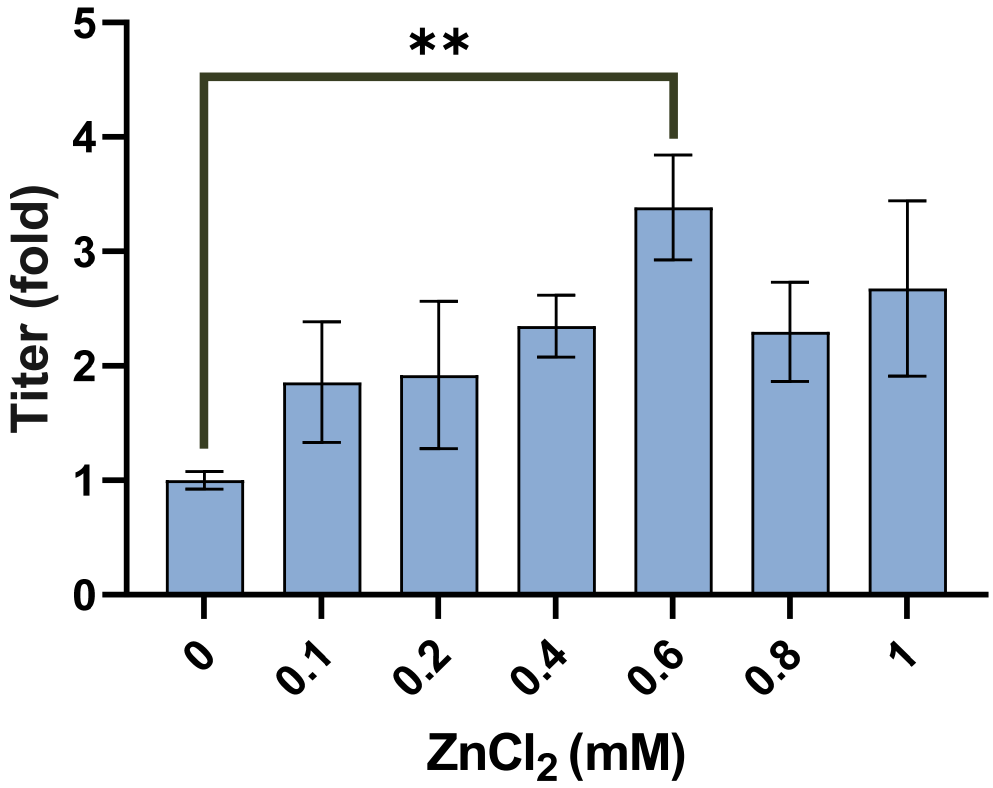

Supplementary Fig. S1. Optimization of metal ions concentration in culture medium for ColH expression. YPD medium containing 10 mM $CaCl_2$ and supplemented with different concentrations of $ZnCl_2$. ** $P < 0.01$.

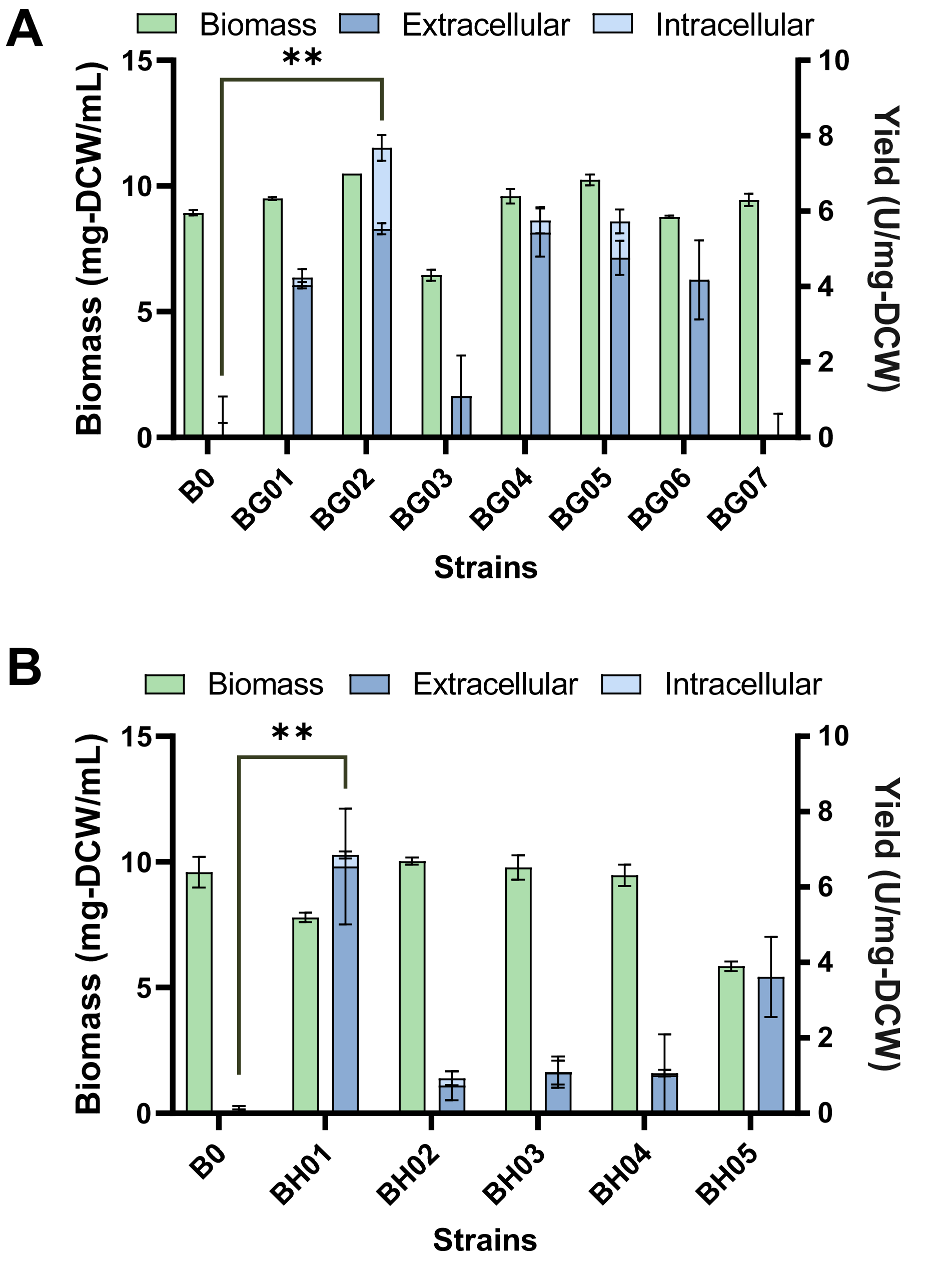

Supplementary Fig. S2.(A) Biomass and collagenase yield of yeast strains B184M expressing ColG; (B) Biomass and collagenase yield by yeast strains B184M expressing ColH; . ** $P < 0.01$.

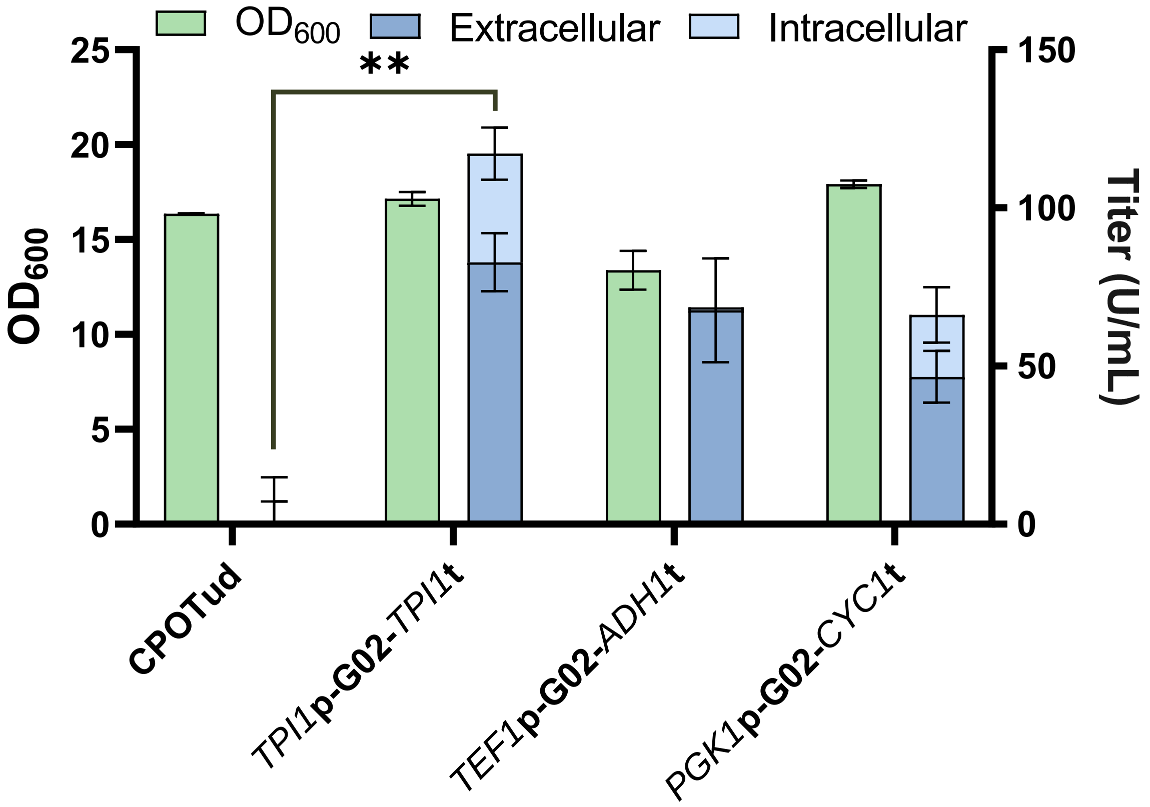

Supplementary Fig. S3. Collagenase expression under control by different protomers and terminators, which replaced the promoter and terminator on the plasmid pCP_G02. Strain B184M was used as a host strain. ** $P < 0.01$.

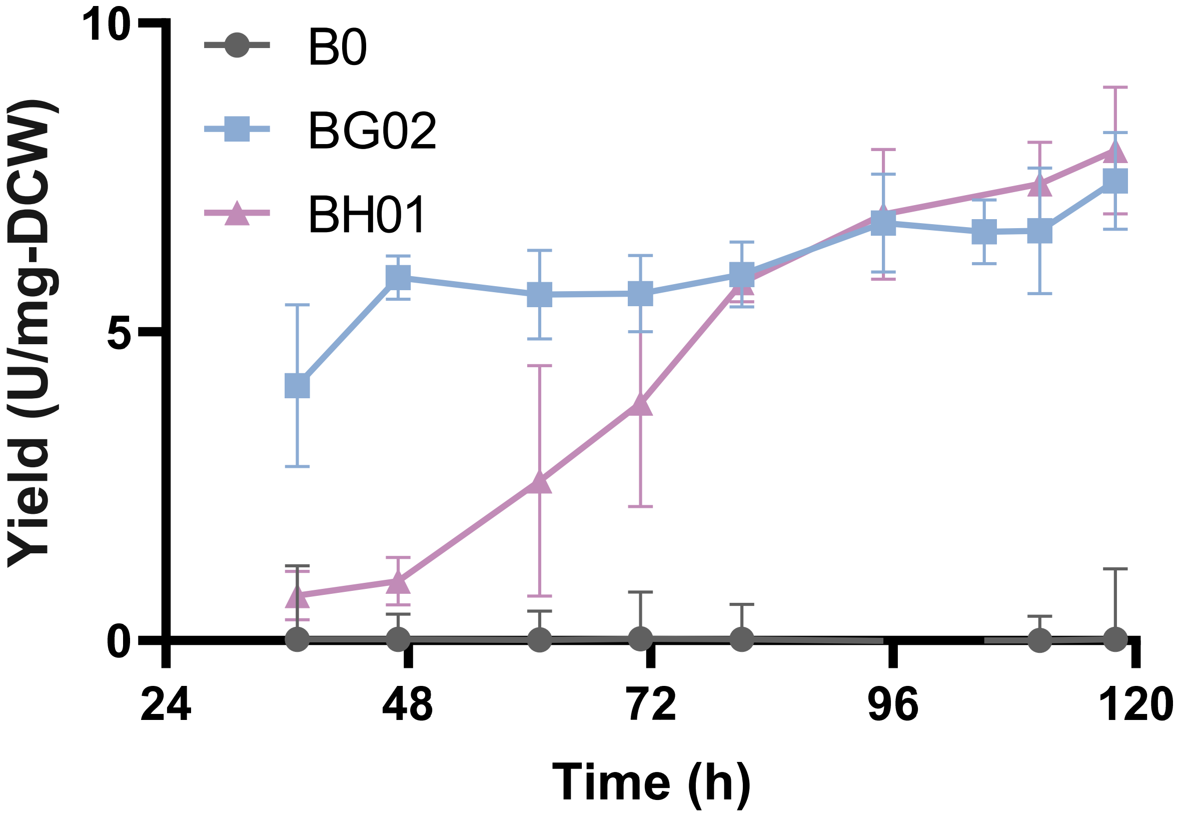

Supplementary Fig. S4. Collagenase yield in batch cultivation of strains BG02, BH01 and the control strain B0.

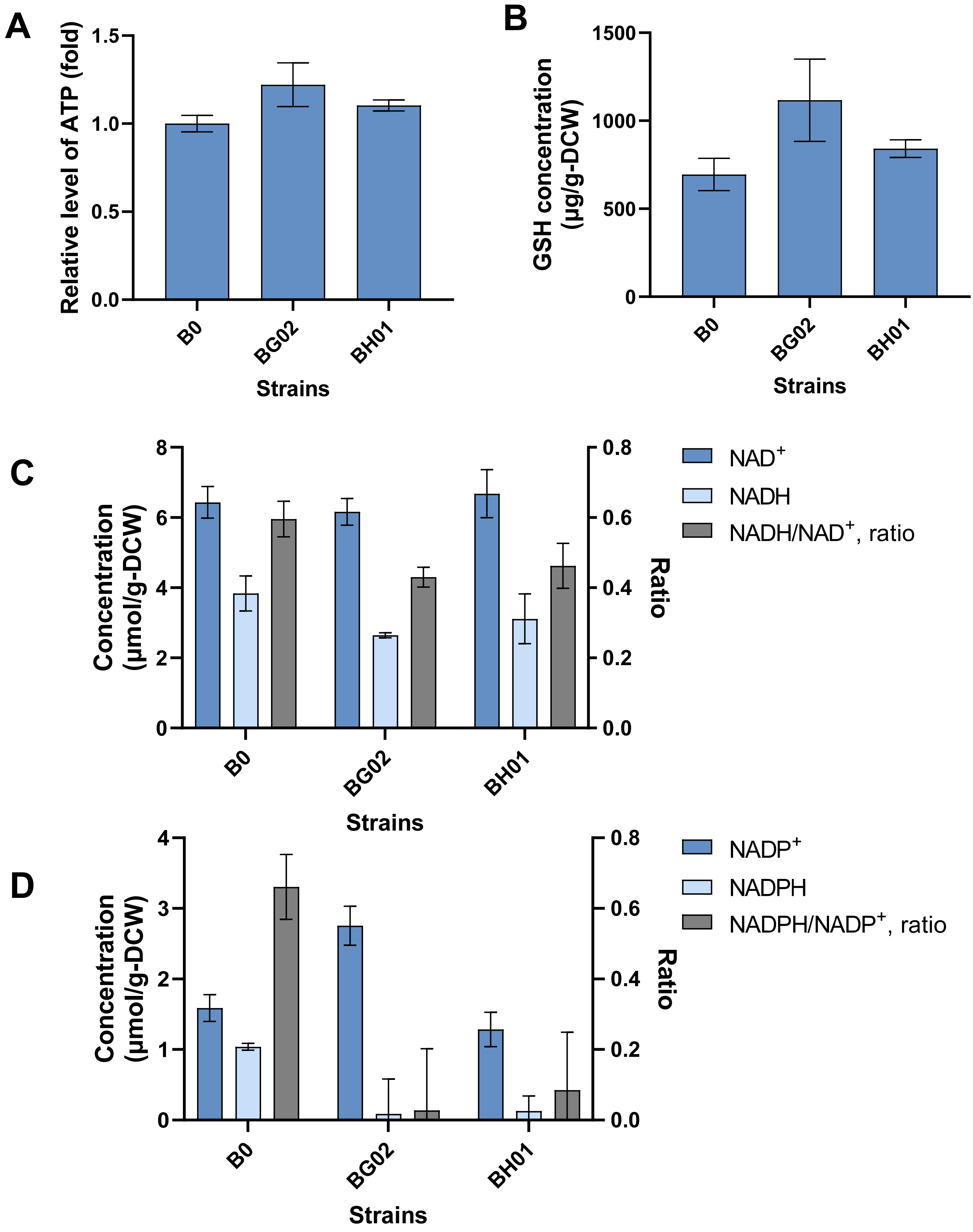

Supplementary Fig. S5. Intracellular cofactor level changed in collagenase expression strains. (A) ATP pool; (B) GSH; (C) NAD^+ and NADH; (D) $NADP^+$ and NADPH. Strains were cultivated in YPD medium supplemented with 10 mM $CaCl_2$ and 0.6mM $ZnCl_2$, and samples were taken from the exponential phase (OD≈1) .

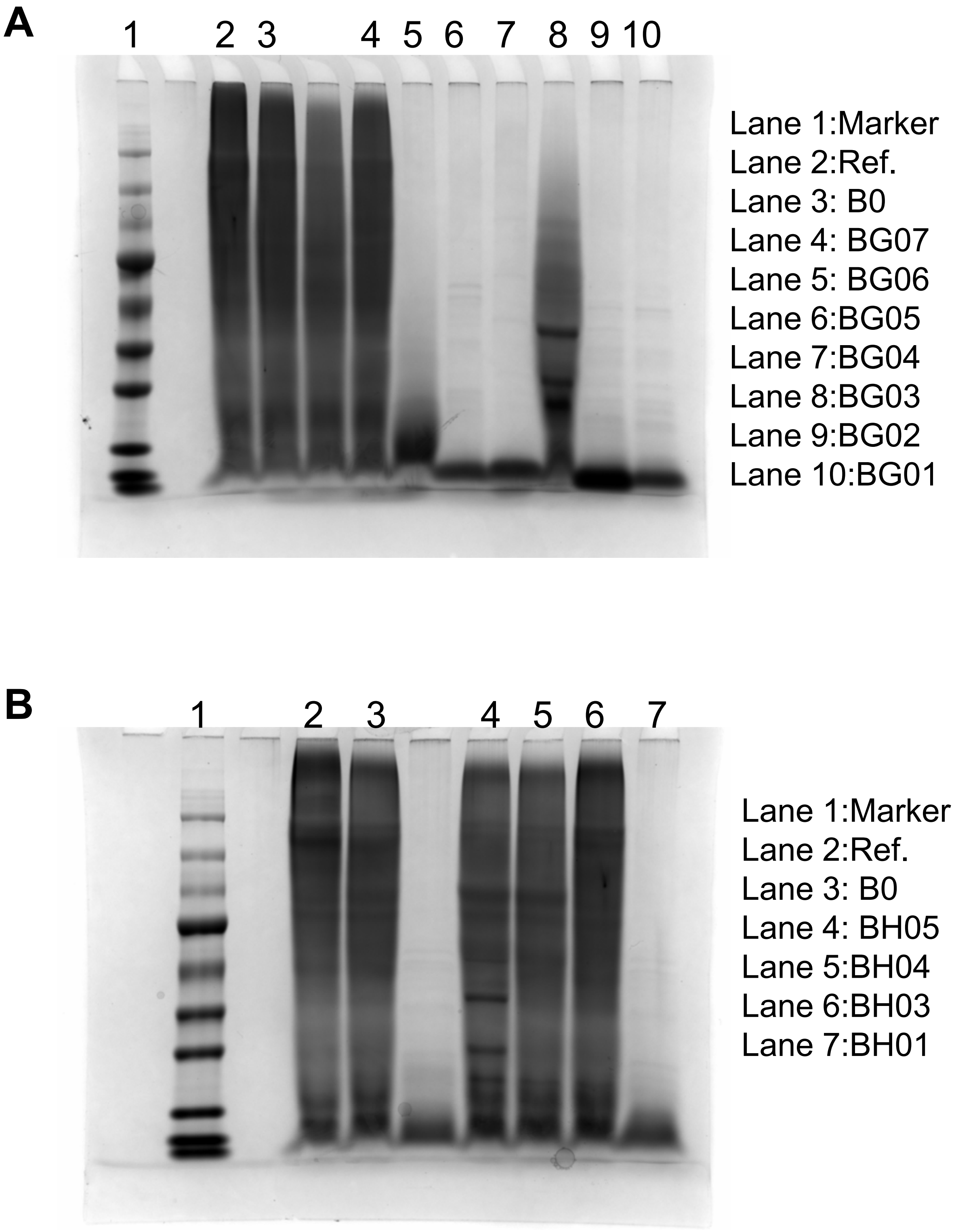

Supplementary Fig. S6. SDS-PAGE analysis of gelatin digestion by recombinant collagenases during cell cultivation. (A) Test for strains expressed ColG; (B) Test for strains expressed ColH. Strains were cultivated in YPD medium with 10 mM $CaCl_2$, 0.6 mM $ZnCl_2$ and 1% gelatin at 30 °C for 96h, then supernatant was used for analysis. Ref.: YPD medium with 10 mM $CaCl_2$, 0.6 mM $ZnCl_2$ and 1% gelatin.

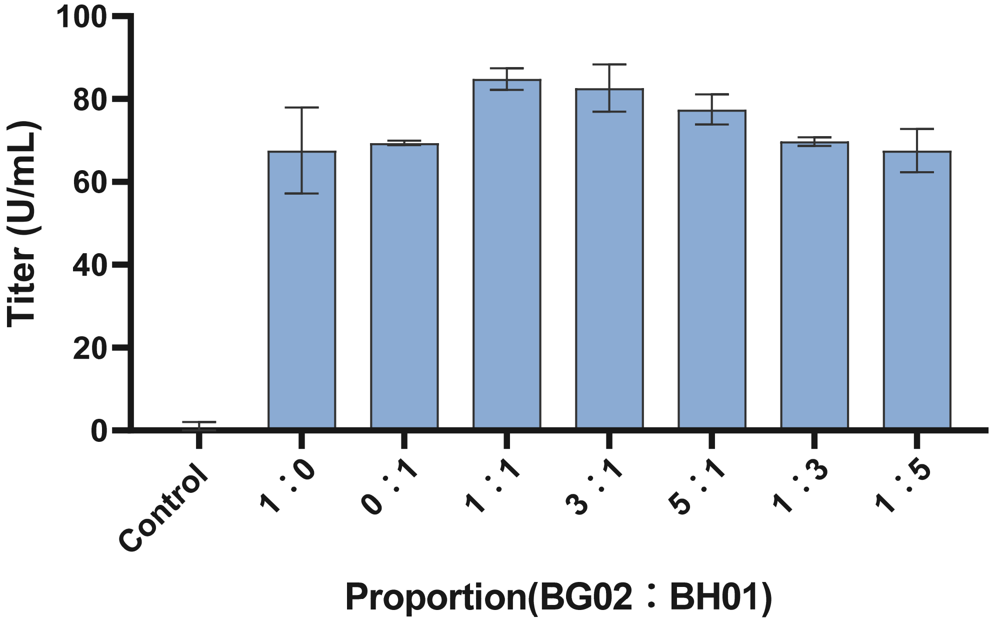

Supplementary Fig. S7. Collagenase activity measurement for the mixture of recombinant ColG and ColH. Strains BG02 and BH01 were cultivated in YPD medium with 10 mM $CaCl_2$ and 0.6 mM $ZnCl_2$ at 30 °C for 96h. Then supernatant was collected and mixed in proportion for activity measurement.

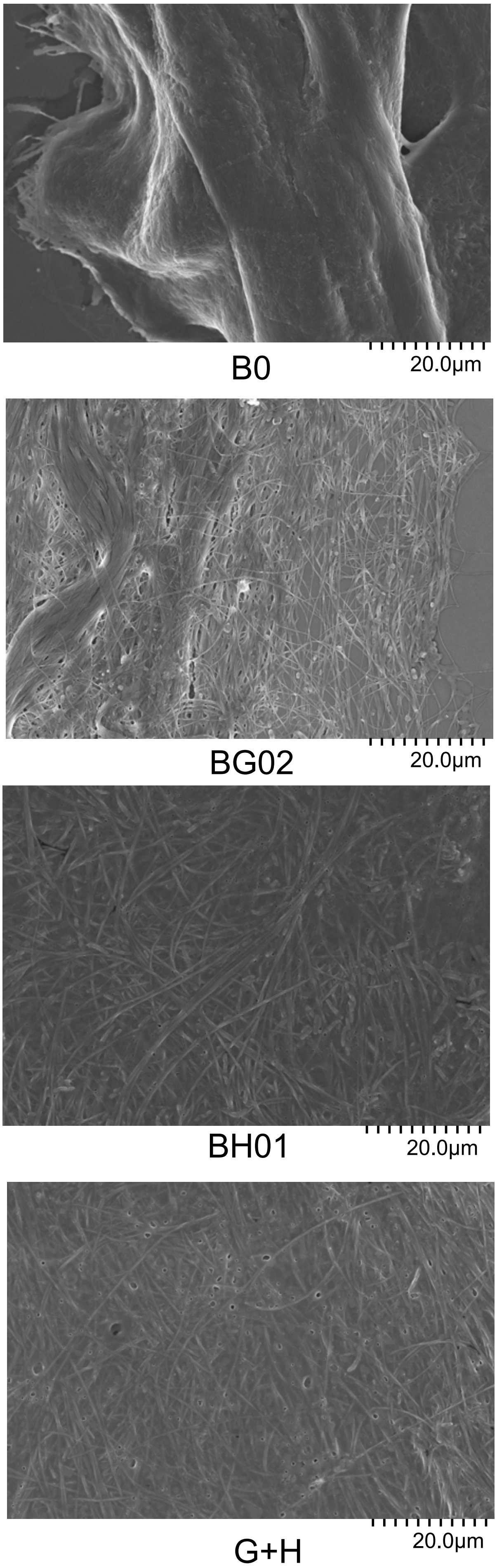

Supplementary Fig. S8. Differences between recombinant ColG and ColH in collagen degradation were revealed at microscopic structure level by using a scanning electron microscope (SEM) with 4000× magnification. Other conditions were the same as in Figure 6C.

Supplementary Table 1 Primers used in this study

Name	Sequence#	Description
Plasmid construction		
GF	CGGGGTACCAACAAAATGAAGAAGAACAT	GF and GR:amplification for *colG* gene with 6×His sequence
GR	GATGCTAGCTTAGTGGTGGTGGTGGTGG CTTGTTAACCCGCAATTCGT	
AP	CTGGGTACCAACAAAATGAGATTTCCATCTA TTTTTACTGCTGTTTTGTTTGCT	add α-factor pre
PG	CTGCTGTTTTGTTTGCTGCTTCTTCTGCTTTG GCTATAGCTAATACAAATTCCG	amplification for *colG* chain including homology arm with α-factor pre
AF	CTGGGTACCAACAAAATGAGATTTCCATCTA T	AF and ARWG:amplification for α-factor leader including homology arm with *colG* chain;ARG:add a spacer after α-factor leader
ARWG	CGGAATTTGTATTAGCTATAGCTTCAGCCTCT CTTTTATCCAAAGAAACACC	
ARG	CGGAATTTGTATTAGCTATTTTTGGTTCACCT TCTTCTC	
WGF	CTGAAGCTATAGCTAATACAAATTCCG	amplification for *colG* chain including homology arm with α-factor leader
AGF	GAAGAAGGTGAACCAAAAATAGCTAATACA AATTCCG	amplification for *colG* chain including homology arm with α-factor leader+spacer
PproG	CTGTTTTGTTTGCTGCTTCTTCTGCTTTGGCT AAGCCAATCGAAAACACTAACG	amplification for *colG* pro-peptide-*colG* chain including homology arm with α-factor pre
ARWproG	CGTTAGTGTTTTCGATTGGCTTAGCTTCAGC CTCTCTTTTATC	amplification for α-factor leader including homology arm with *colG* pro-peptide;ARproG:add a spacer after α-factor leader
ARproG	TTAGTGTTTTCGATTGGCTTTTTGGTTCACC TTCTTCTCTTTTAT	
WproGF	GATAAAAGAGAGGCTGAAGCTAAGCCAATC GAAAACACTAACG	amplification for *colG* pro-peptide-*colG* chain including homology arm with α-factor leader
proGF	GAGAAGAAGGTGAACCAAAAAAGCCAATC GAAAACACTAACG	amplification for *colG* pro-peptide-*colG* chain including homology arm with α-factor leader+spacer
HF	CGGGGTACCAACAAAATGAAGAGAAAATG	HF and HR:amplification for *colH* gene with 6×His sequence
HR	GATGCTAGCTTAGTGGTGGTGGTGGTGG TCTACCAACAGAACCTTCAATG	
ARWH	GGATTCGTTTTGGACAGCTTCAGCCTCTCTT TTATCCAAAGAAACACCTTC	amplification for α-factor leader including homology arm with *colH*

ARH	CGCTTGGATTCGTTTTGGACTTTTGGTTCACCTTCTTC	chain;ARH:add a spacer after α-factor leader
WHF	GAAGCTGTCCAAAACGAATCCAAGCGTTAC	amplification for *colH* chain including homology arm with α-factor leader
AHF	GAAGGTGAACCAAAAGTCCAAAACGAATCCAAGCG	amplification for *colH* chain including homology arm with α-factor leader+spacer
ARWproH	CATTGTTCTTGTCAACAGCAGCTTCAGCCTCTCTTTTATC	amplification for α-factor leader including homology arm with *colH*
ARproH	GCATTGTTCTTGTCAACAGCTTTTGGTTCACCTTCTTCTCTTTTAT	pro-peptide; ARproH:add a spacer after α-factor leader
WproHF	GATAAAAGAGAGGCTGAAGCTGCTGTTGACAAGAACAATG	amplification for *colH* pro-peptide-*colH* chain including homology arm with α-factor leader
proHF	GAGAAGAAGGTGAACCAAAAGCTGTTGACAAGAACAATGCC	amplification for *colH* pro-peptide-*colH* chain including homology arm with α-factor leader+spacer
P_TEF1	AAGCGGCCGCAACAAAATGAGATTTC	
T_ADH1	CGAGCTCTTAGTGGTGGTGGTGGTGGTG	
CpTEF1	CGATAGCAACCGTTGGCATGGATCCGGCCGGCCGCACACACCA	for promoter and terminator replacement, amplification for *TEF1*p-Col-*ADH1*t
CtADH1	TATAATGTTACATGCGTACACGCGTGAGCGACCTCATGCTATACCTGAGAAAGCAACC	
P_PGK1	AAGGATCCAACAAAATGAGATTTCCATC	
T_CYC1	CTTATGCTAGCTTAGTGGTG	
CpPGK1	CGATAGCAACCGTTGGCATGGATCCGGCCGGCCTGGAAGTACC	for promoter and terminator replacement, amplification for *PGK1*p- Col-*CYC1*t
CtCYC1	TATAATGTTACATGCGTACACGCGTCTTCGAGCGTCCCAAAACCTTC	
verification		
G1	ATGTTGTCTTCCACGGTGTTTT	for ColG expression plasmids
G2	CCTTGTCAGAGGAGTCATTGTTTT	
H1	CATCGGTAGCGTAGGAGACAGC	for ColH expression plasmids
H2	CCAAACGAAGGTGATTCCAAGA	

Underlined sequence indicates restriction site.
